# How uro-oncology has been affected by COVID-19 emergency? Data from Piedmont/Valle d’Aosta Oncological Network, Italy

**DOI:** 10.1177/0391560320946186

**Published:** 2021-02-25

**Authors:** Marco Oderda, Giorgio Calleris, Marco Falcone, Giuseppe Fasolis, Giovanni Muto, Gianluca Oderda, Francesco Porpiglia, Alessandro Volpe, Oscar Bertetto, Paolo Gontero

**Affiliations:** 1 Division of Urology, Molinette Hospital—Città della Salute e della Scienza di Torino, University of Turin, Torino, Italy; 2 Division of Urology, Ospedale San Lazzaro, ASL-CN2 Alba-Bra, Alba, Italy; 3 Humanitas Gradenigo Hospital, Torino, Italy; 4 Credit Suisse AG, Zurich, Switzerland; 5 Division of Urology, University of Turin, San Luigi Gonzaga Hospital, Orbassano, Italy; 6 Maggiore della Carità Hospital, University of Eastern Piedmont, Novara, Italy; 7 Rete Oncologica del Piemonte e della Valle d’Aosta, Torino, Italy

**Keywords:** COVID, coronavirus, cancer, urology, Piedmont

## Abstract

**Introduction:**

Coronavirus disease 2019 (COVID-19) pandemic has dramatically hit all Europe and Northern Italy in particular. The reallocation of medical resources has caused a sharp reduction in the activity of many medical disciplines, including urology. The restricted availability of resources is expected to cause a delay in the treatment of urological cancers and to negatively influence the clinical history of many cancer patients. In this study, we describe COVID-19 impact on uro-oncological management in Piedmont/Valle d’Aosta, estimating its future impact.

**Methods:**

We performed an online survey in 12 urological centers, belonging to the Oncological Network of Piedmont/Valle d’Aosta, to estimate the impact of COVID-19 emergency on their practice. On this basis, we then estimated the medical working capacity needed to absorb all postponed uro-oncological procedures.

**Results:**

Most centers (77%) declared to be “much”/“very much” affected by COVID-19 emergency. If uro-oncological consultations for newly diagnosed cancers were often maintained, follow-up consultations were more than halved or even suspended in around two out of three centers. In-office and day-hospital procedures were generally only mildly reduced, whereas major uro-oncological procedures were more than halved or even suspended in 60% of centers. To clear waiting list backlog, the urological working capacity should dramatically increase in the next months; delays greater than 1 month are expected for more than 50% of uro-oncological procedures.

**Conclusions:**

COVID-19 emergency has dramatically slowed down uro-oncological activity in Piedmont and Valle d’Aosta. Ideally, uro-oncological patients should be referred to COVID-19-free tertiary urological centers to ensure a timely management.

## Introduction

Since the first report in China, severe acute respiratory syndrome coronavirus 2 has rapidly spread worldwide, and coronavirus disease 2019 (COVID-19) has been declared a pandemic by the World Health Organization (WHO) on 11 March 2020.^1^ Italy has been among the first European countries to be affected and one of the worst hit. As of 27 April 2020, there are 199,414 confirmed positive cases of COVID-19, and 26,977 deaths.^2^ The north of Italy, in particular, has taken the blow, with Lombardy, Piedmont, and Emilia-Romagna being the regions with the higher number of cases and deaths. Despite the containment measures introduced by the national government and health authority, the situation is not yet under control. The rapid surge of COVID-19 patients made necessary the reallocation of medical resources to face this crisis. As a consequence, the level of activity of all medical disciplines not primarily involved in the management of COVID-19 patients, including urology, has dramatically reduced.^3^

Given the need to increase bed allowance for COVID-19 admissions, to free lifesaving equipment such as ventilators, to free medical personnel, and to minimize the number of patients physically attending into the hospital, the urological activity has been limited to urgent procedures or oncological priorities.^4^ Despite several recommendations have been published in the last weeks as a guidance to prioritize surgeries, what is to be really considered as a uro-oncological priority still remains subject of debate.^3–7^ What is clear to uro-oncologists is that the restricted availability of operatory rooms (OR) will inevitably cause a delay in the surgical treatment of urological cancers. In long-term settings, considering that the duration of the crisis is prolonged and unpredictable, this issue may potentially harm patients more than the COVID-19 itself.^8^

The aim of this study was to provide data on the current uro-oncological management in the main centers belonging to the Oncological Network of Piedmont/Valle d’Aosta, Italian regions among the most affected by COVID-19, estimating its future impact on surgical planning.

## Methods

Twelve urological centers belonging to the Oncological Network of Piedmont/Valle d’Aosta were included in the current study. A description of each center is provided in Table 1. A survey was performed using SoGoSurvey platform (SoGoSurvey, Herndon, VA, USA): urologists were asked to report their surgical activity on prostate, bladder, and kidney cancer from 1 to 31 March 2020, as compared to a regular month before COVID-19 outbreak.

**Table 1.** Participating institutions within the Oncological Network of Piedmont/Valle d’Aosta.

Center	City	Region	Hospital type
Ospedale San Lazzaro, ASL-CN2	Alba-Bra	Piedmont	Non academic, public
Ospedale Regionale Valle d’Aosta Umberto Parini	Aosta	Valle d’Aosta	Non academic, public
Nuovo Ospedale degli Infermi, ASL Biella	Biella	Piedmont	Non academic, public
ASL-TO4	Cirié-Chivasso- Ivrea	Piedmont	Non academic, public
Azienda Ospedaliera Santa Croce e Carle	Cuneo	Piedmont	Non academic, public
Azienda Ospedaliera Universitaria Maggiore della Carità	Novara	Piedmont	Academic, public
Ospedale San Giacomo	Novi Ligure	Piedmont	Non academic, public
Azienda Ospedaliera Universitaria San Luigi Gonzaga	Orbassano	Piedmont	Academic, public
ASL-TO3	Rivoli-Collegno-Pinerolo	Piedmont	Non academic, public
Azienda Ospedaliera Ordine Mauriziano di Torino	Torino	Piedmont	Non academic, public
Azienda Ospedaliera Universitaria Città della Salute e della Scienza di Torino, Presidio Molinette	Torino	Piedmont	Academic, public
Ospedale Humanitas Gradenigo, Humanitas University di Rozzano	Torino	Piedmont	Academic, private within national health system

The items addressed in the survey were as follows:

The estimated impact of COVID-19 emergency on the uro-oncological practice;
The estimated reduction (%) of uro-oncological consultations for newly diagnosed cancers and follow-up visits;
The estimated number (%) of uro-oncological surgeries (prostate biopsies, TURBs, radical prostatectomies, radical cystectomies, radical/partial nephrectomies, nephroureterectomies, robotic surgeries) in March as compared to a regular month before COVID-19 outbreak;
The estimated percentage of uro-oncological procedures having a delay beyond 30 days in waiting list due to COVID-19 emergency;
The type of uro-oncological surgeries performed at each institution during March.

Given similar reduction rates from normal activity to what seen in March, we tried to estimate the medical working capacity which would be needed in order to absorb uro-oncological interventions which have been postponed second to the pandemic. The result has been derived by randomly simulating monthly reductions in each type of intervention, as well in their total, based on the statistical distribution of survey reduction rates. Statistical analyses were performed with Microsoft Excel 10 (Microsoft Corporation, Redmont, WA, USA).

## Results

**Figure 1.**
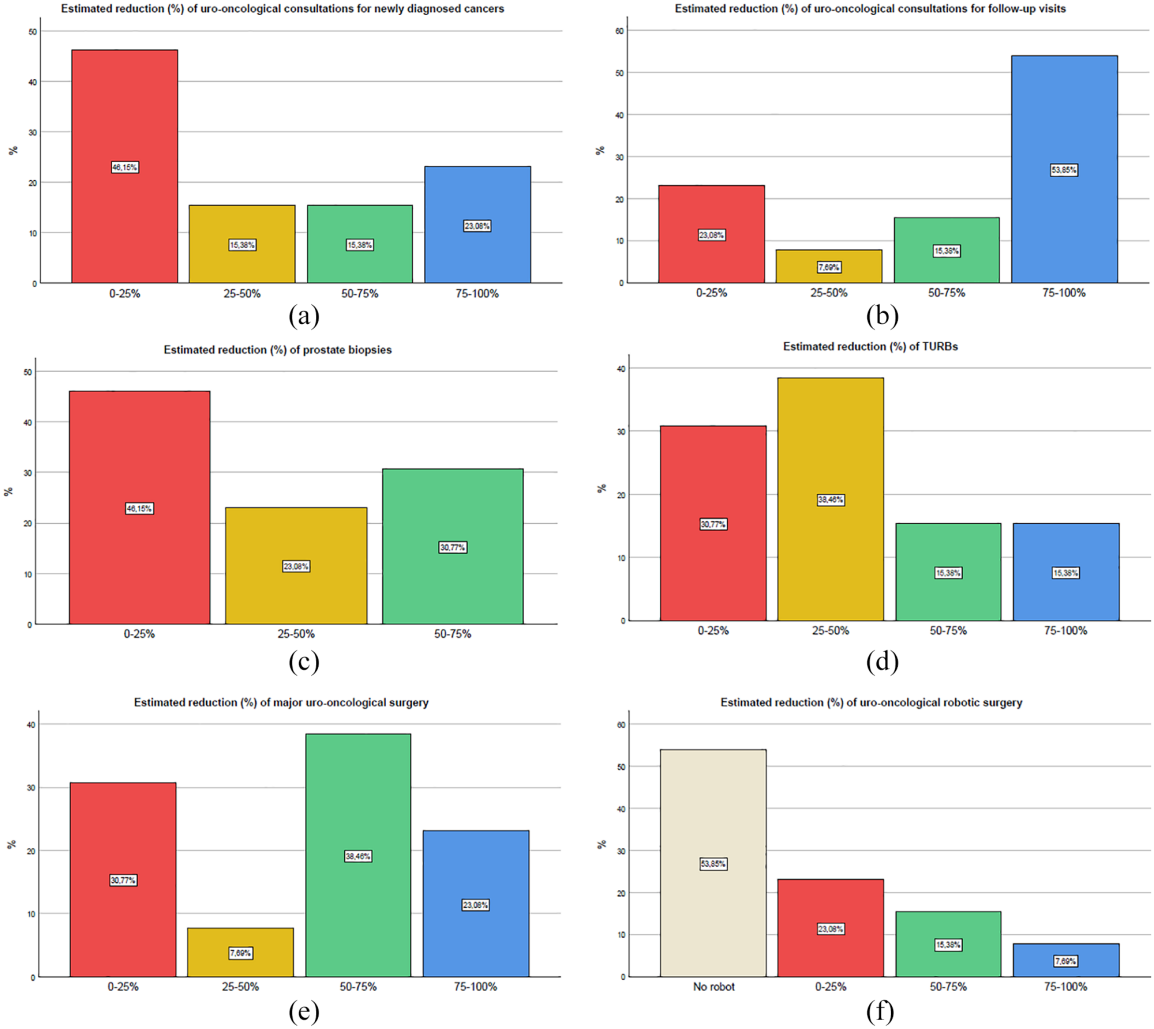
Reduction in uro-oncological activities during March 2020.

**Figure 2.**
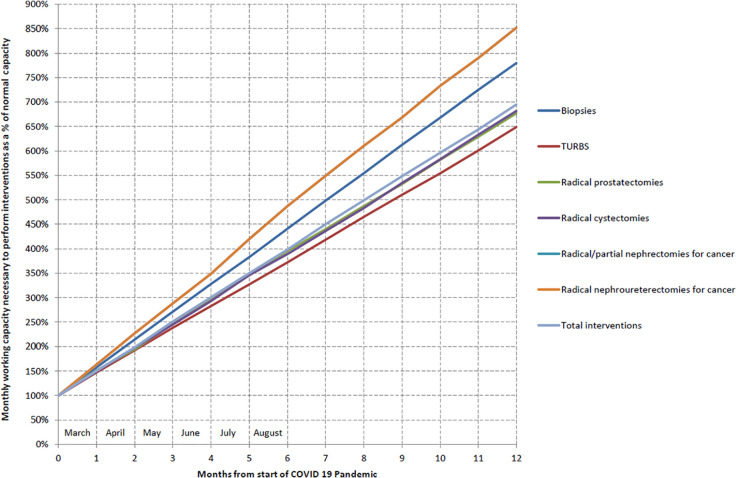
Estimation of the medical working capacity needed to absorb uro-oncological interventions as a function of pandemic duration.

Overall, most centers (77%) declared to be “much” or “very much” affected by COVID-19 emergency in the uro-oncological management. Figure 1 shows the estimated reduction of main activities. Uro-oncological consultations for newly diagnosed cancers were not significantly restricted in the majority of centers, while the opposite was noted for follow-up consultations that were more than halved or even suspended in more than 70% of centers. Prostate biopsies were reduced by 57%, with a mean decrease of 15 biopsies/month, while transurethral resection of the bladder (TURBs) underwent a smaller but still significant restriction of 49%, with a mean decrease of 12 procedures/month. Around 70% of centers reported an effort to maintain the availability of these basic uro-oncological procedures that can be performed in-office or in a day-hospital setting, with a restriction less than 50%. As for major uro-oncological procedures, centers reported a dramatic reduction of activity, being more than halved or even suspended in 60% of centers. On average, radical prostatectomies, radical cystectomies, radical/partial nephrectomies, and nephroureterectomies sustained decrease by 49%, 49%, 45%, and 62%, respectively. Six centers with availability of Da Vinci robot tried to maintain their robotic activity, following the recent recommendations on minimally invasive surgery.^5^ Table 2 shows the decrease in each procedure during the month of March. The estimation of the urological working capacity needed to absorb uro-oncological interventions which have been postponed second to the pandemic shows that surgical procedures should dramatically increase in the next months to treat all patients accumulated on waiting list (Figure 2). More than 50% of uro-oncological procedures are estimated to have a delay beyond 30 days on waiting list.

**Table 2.** Estimated decrease in uro-oncological procedures due to COVID-19 emergency in March 2020.

Procedure	Estimated decrease in March
Prostate biopsy	57%
TURB	45%
Radical prostatectomy	49%
Radical cystectomy	49%
Radical/partial nephrectomy	45%
Nephroureterectomy	62%

## Discussion

The north of Italy, including Piedmont, has been one of the regions worst hit by COVID-19, with 25,098 confirmed cases and 15,508 deaths as of 27 April 2020. Strict containment measures have been introduced by the Italian government since 23 February, with the aim to slow down the contagion.^9^ Among these measures, health authorities have demanded the hospitals to hold on all non-urgent elective procedures, in order to dedicate more resources to the management of COVID-19 patients. As for urologists, this has meant that only urgent conditions (such as testicular torsions or obstructive uropathy) and non-deferrable oncologic diseases could be treated. Several recommendations have been recently published to aid the scheduling of surgeries.^3,10^ A five-point scale for surgical priority tiers has been developed by Cleveland Clinic, ranging from “score 0” emergency (i.e. testicular torsion) to “score 4” non-essential procedures (i.e. living donor transplantation).^10^ Ficarra et al.^3^ have divided the urological cancer surgeries into four categories: (1) non-deferrable, including all procedures whose delay can jeopardize cancer-related outcomes; (2) semi-non-deferrable, to be considered in regions with limited diffusion of COVID-19; (3) deferrable; and (4) replaceable by other treatments, such as radiation therapy or chemotherapy.

What is to be considered as a uro-oncological priority, however, remains debatable.

In Piedmont and Valle d’Aosta, our Oncological Network monitors the management of oncological patients with the aim to guarantee a high standard of care to all patients. One of its main achievements is the rapid process for the diagnosis, staging, and treatment of oncological diseases. We find most alarming that uro-oncological activity has been dramatically reduced due to COVID-19 emergency, with more than 50% of uro-oncological procedures estimated to exceed the recommended time-frame of 30 days within which newly diagnosed cases should be definitely treated. This is of particular concern for several reasons:

The reduction in the number of diagnostic exams and consultations will lead to underdiagnose many urological cancers, which might show up in the next months, possibly in more advanced stages;
The postponement of uro-oncological surgeries could affect the oncologic and/or functional outcomes, without considering the implications in terms of patient anxiety and related depression. Furthermore, in the era of tailored therapy, we find it hard to consider a surgery replaceable by other treatment only because of the COVID-19 pandemic;
The reduction in uro-oncological surgeries involved also the treatment of aggressive cancers such as urothelial neoplasms of the bladder or the upper tract, which have been successively classified among non-deferrable, high-priority surgeries.^11,12^ We have to consider, though, that the survey is referred to March, when the emergency reached its peak;
The delay in consultations and surgeries registered in March will have a ripple effect with a delay of future patients who will additionally suffer of longer waiting lists, as shown in our graph. Given that consultation and surgical volumes of each institution cannot increase beyond a certain point, health system will have to develop strategies to catch up with these delays when the pandemic will be over.

All these things considered, we believe that in time of crisis, uro-oncological consultations and surgeries should be centralized in tertiary urological centers that should ideally remain COVID-19-free sanctuaries, or at least maintain unchanged their uro-oncological activity thanks to COVID-19-free paths through the hospital.^13^ This way the accumulation of delay would be limited, if not avoided at all, while assuring oncological patients high-quality, timely, and safe treatments, including robotic surgery that equipped centers struggle to maintain.

This study is affected by several limitations, providing only estimates of activity in a relatively small number of centers. Furthermore, there was heterogeneity in participating institutions, including both academic and non-academic centers with different resources and surgical volumes. However, we provided a firsthand snapshot of the uro-oncological management in one of the Italian regions most deeply hit by COVID-19, highlighting dangerous indirect effects of the pandemic that could become more harmful than the virus itself.

## Conclusion

Due to COVID-19 emergency, all major uro-oncological procedures in Piedmont and Valle d’Aosta reported a dramatic reduction, as well as prostate biopsies and TURBs. Uro-oncological consultations were mostly reduced with the exception of these for newly diagnosed cancers. To guarantee a high-quality and timely management, uro-oncological patients should ideally be addressed to COVID-19-free tertiary urological centers.
